# Evaluation of polyphenylene ether ether sulfone/nanohydroxyapatite nanofiber composite as a biomaterial for hard tissue replacement

**DOI:** 10.1186/2194-0517-2-2

**Published:** 2013-02-06

**Authors:** Manickam Ashokkumar, Dharmalingam Sangeetha

**Affiliations:** Department of Chemistry, Anna University, Sardar Patel Road, Chennai, Tamil Nadu 600025 India

**Keywords:** Polyphenylene ether ether sulfone, Nanohydroxyapatite, Bioactivity, Biomineralization, Osteoblast cells

## Abstract

The present work is aimed at investigating the mechanical and *in vitro* biological properties of polyphenylene ether ether sulfone (PPEES)/nanohydroxyapatite (nHA) composite fibers. Electrospinning was used to prepare nanofiber composite mats of PPEES/nHA with different weight percentages of the inorganic filler, nHA. The fabricated composites were characterized using Fourier transform infrared spectroscopy (FTIR)-attenuated total reflectance spectroscopy (ATR) and scanning electron microscopy (SEM)-energy dispersive X-ray spectroscopy (EDX) techniques. The mechanical properties of the composite were studied with a tensile tester. The FTIR-ATR spectrum depicted the functional group as well as the interaction between the PPEES and nHA composite materials; in addition, the elemental groups were identified with EDX analysis. The morphology of the nanofiber composite was studied by SEM. Tensile strength analysis of the PPEES/nHA composite revealed the elastic nature of the nanofiber composite reinforced with nHA and suggested significant mechanical strength of the composite. The biomineralization studies performed using simulated body fluid with increased incubation time showed enhanced mineralization, which showed that the composites possessed high bioactivity property. Cell viability of the nanofiber composite, studied with osteoblast (MG-63) cells, was observed to be higher in the composites containing higher concentrations of nHA.

**Electronic supplementary material:**

The online version of this article (doi:10.1186/2194-0517-2-2) contains supplementary material, which is available to authorized users.

## Background

The term biomaterial, which is closely related to applications that repair or replace a part of or the whole tissue, must have favorable mechanical as well as biological properties and should play a major role in developing successful implantations by means of inducing cell adhesion, proliferation, and differentiation on the surface of the biomaterial (Sista et al. 2011). In developing new biomaterials for tissue replacement, the structure and properties of the tissue which is to be replaced, i.e., the biological template, must be taken into consideration. This is because, if properties of the new material are significantly different from those of the host tissue, the material under development will cause dynamic changes to the host tissue after implantation and thus will not achieve the goals embedded in the original conceptual design (Wang 2003). Different materials have been investigated for applications in bone tissue engineering—metals, ceramics, and polymers. As with all materials implanted into the body, the polymers for bone regeneration must be biocompatible. In addition, they should be moldable, shapeable, or polymerizable *in situ* to ensure good integration in the defective area (Seal et al. 2001). However, the polymer materials used for orthopedic application do not exhibit adequate mechanical properties and bioactive behavior, which are the main disadvantages for bone tissue engineering. In order to overcome these problems, polymer/bioactive ceramic composites have been developed for bone tissue engineering, which ensure the achievement of the above-mentioned properties and performance of the material (Ryszkowska et al. 2010).

It is well known that the two fundamental factors to be considered in producing polymer nanocomposites with bone-like properties are (1) good interfacial adhesion between organic polymers and inorganic hydroxyl apatite (HA) and (2) uniform dispersion of HA at the nanolevel in the polymer nanofiber (Lee et al. 2007). When such a composite is immersed in simulated body fluid (SBF), biologically active HA layers are formed on the implant due to the ion-exchange reaction between the bioactive implant and the surrounding body fluids which is chemically and crystallographically equivalent to the mineral phase of the bone (Pielichowska and Blazewicz 2010). In addition, HA is known to smartly utilize the apatite that is mineralized on their surfaces as an interface to integrate spontaneously with the living tissue (Kim et al. 2004). Nanofiber composites have certain favorable characteristics and properties, such as porosity, the surface area-to-volume ratio, pore size, pore interconnectivity, structural strength, and biocompatibility, which play a major role in the design and fabrication of polymeric materials for bone tissue engineering (Tan et al. 2003; Teoh 2004).

Although existing bioactive materials possess high compressive strength, they are unfortunately very brittle and have inherently poor tensile and torsional properties. Material selection is especially important in bone tissue engineering because a supporting substrate is critical in maintaining mechanical strength and structural support as well as providing the optimal culturing environment for bone formation during the early stages of the regenerative process (Lu et al. 2003). The large surface area-to-weight ratio of the composite material offered by electrospinning (Reneker and Chun 1996; Li and Xia 2004; Darrell et al. 2006; Ramakrishna et al. 2006; Greiner and Wendorff 2007) is achieved by means of decreasing the diameter of the fiber from the micrometer (10–100 μm) to submicron or nanometer level (10 × 10^−3^ to 100 × 10^−3^), resulting in the appearance of several amazing characteristics such as flexibility in surface functionalities and superior mechanical properties (stiffness and tensile strength) compared with any other known form of material (Huang et al. 2003).

The present study is focused on designing and developing the polymer—polyphenylene ether ether sulfone (PPEES) nanofiber composites reinforced with nanohydroxyapatite (nHA)—and evaluating its potential application as an orthopedic biomaterial. The prepared nanofiber composite was subjected to characterization and morphology studies using Fourier transform infrared (FTIR)-attenuated total reflectance spectroscopy (ATR) and scanning electron microscopy (SEM)-energy dispersive X-ray spectroscopy (EDX) to identify the presence of a structural group and morphology of the composites, respectively. Inverted fluorescence microscopy was used to identify the viability of bone-like cells over the nanofiber composite. The composite was then investigated *in vitro* for its multifunctional properties (mechanical and biological properties) before and after incubation in SBF in order to evaluate the compatibility of the biomaterial for orthopedic application.

## Results and discussion

### FTIR-ATR

**Figure 1:**
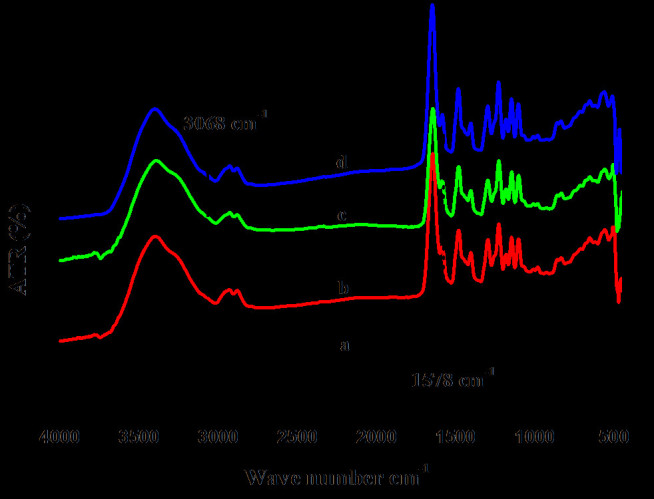
**FTIR-ATR spectra of PPEES nanofiber and its composite: (** ***curve a*** **) PPEES nanofiber, (** ***curve b*** **) PPEES 1, (** ***curve c*** **) PPEES 2, and (** ***curve d*** **) PPEES 3.**

The FTIR-ATR spectra of nHA reinforced PPEES nanofiber composites and bare nanofiber mat are shown in Figure 1. From the spectra, the intense broad band (Figure 1b–d) observed at 3500 cm^−1^ was assigned to the OH stretching vibration which was mainly observed when steric hindrance prevents polymeric association and also confirmed the interaction of nHA with PPEES. The intensity of the peak at 3068 cm^−1^ (Figure 1a) was due to the fact that the adsorption peak of C-H stretching vibration was overlapped by the O-H stretching vibration peaks of nHA. The C=C aromatic ring vibrations were attributed to the peaks occurring at 1578 cm^−1^; intensity of the peak decreased with the addition of nHA due to the interaction of HA with the polymer backbone. The peak at 1375 cm^−1^ corresponds to the ester linkage of the polymer chain. The strong absorption peaks just above 1250 and 1100 cm^−1^ correspond to the diaryl sulfone (Ar-SO_2_-Ar) and diaryl ether (Ar-O-Ar) groups, respectively (Dahe et al. 2011). It was observed that the phosphate vibrations were merged with the S=O vibrations just above 1000 cm^−1^. The aromatic ring CH bending vibration occurred just above 800 cm^−1^.

### Morphology

**Figure 2:**
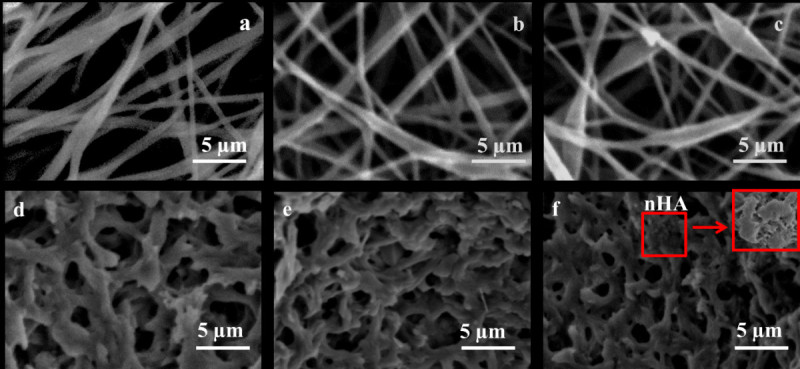
**SEM images showing surface morphology (a–c) and cross-sectional images (d–f) of PPEES and its composite.** (**a**) PPEES, (**b**) PPEES 2, (**c**) PPEES 3, (**d**) PPEES, (**e**) PPEES 2, and (**f**) PPEES 3.

The surface morphology of PPEES and its nanofiber composite with different weight percentages are shown in Figure 2. The SEM image showed the nanofiber in the range of approximately 100–150 nm in diameter, which would provide high surface area-to-weight ratio than any other form of material such as films or membranes. The composite material with high surface area supported apatite formation, cell adhesion, proliferation, and differentiation of bone-like cells. Non-reinforced PPEES showed good fiber formation (Figure 2a) without the formation of beads. SEM analysis of the nHA reinforced composites (PPEES 1, 2, and 3) revealed that at higher filler concentrations, the bead formation increased. In comparison, PPEES 1 (figure not shown) and PPEES 2 (Figure 2b) showed better fiber formation than PPEES 3 (Figure 2c), and random dispersion of nHA in the PPEES nanofiber matrix was observed. In PPEES 3, some beads were observed in between the nanofibers with respect to the increased weight percentage of nHA (Figure 2c). In addition, further evidence for nHA encapsulation within the polymer nanofiber matrix was confirmed with the cross-sectional image of the nanofiber composite (Figure 2d–f). The inset in Figure 2f shows evidence for the presence of nHA. Though both PPEES 1 and PPEES 2 showed good fiber mat formation, PPEES 2 was chosen over PPEES 1 for the rest of the *in vitro* studies since it contained the higher percentage of nHA.

### Mechanical properties

Tensile strength of the PPEES and PPEES 2 nanofiber composites are represented in Table 1. While it was noted that the PPEES nanofiber mat possessed low levels of stiffness, as inferred from Table 1, the incorporation of the inorganic filler into the PPEES nanofiber matrix enhanced the percentage of elongation with slight reduction in tensile strength, which was concurrent with earlier studies (Salerno et al. 2010). The increase in percentage of elongation was explained by considering that the HA particles in the continuous phase of the polymer matrix constituted a second phase. The interface between the two phases (PPEES and nHA) acts like a grain boundary, resisting the propagation of a crack when subjected to tensile forces. Rather, a friction force that is opposite to the direction of the crack-initiating force will be present at the edges of the crack, leading to the observed increase in elongation (Zebarjad et al. 2011). When developing a biomaterial for orthopedic application, due importance shall be given to the mechanical properties of the material because most of the polymeric materials, even if they possess good biocompatibility, fail to withstand *in vivo* stresses due to their brittle nature. The fabrication of composite materials reinforced with fillers is one way of avoiding the problems of brittleness. The property transition from being brittle to ductile of the composite was achieved by the incorporation of the inorganic filler, resulting in an enhanced mechanical strength of the composite (Broza et al. 2005; Bunsell and Renard 2005; Bismarck et al. 2001).

**Table 1:** **Tensile strength and percentage elongation of the nanofiber composites**

Composites Studied	Tensile strength (MPa)	Elongation (%)
PPEES	56.32	33.25
PPEES2	52.26	42.05
PPEES2^a^	49.17	44.74
PPEES2^b^	48.26	46.21
PPEES2^c^	47.78	52.31

^a^10 days of incubation with SBF; ^b^15 days of incubation with SBF; ^c^30 days of incubation with SBF.

Furthermore, the mechanical properties of the composite materials were studied with the percentage elongation obtained from the tensile tests (Table 1). Substantial increased elongation on PPEES 2 nanofiber composite was observed when compared with the bare PPEES nanofiber mat. Also, the percentage elongation of PPEES 2 was increased significantly with respect to time when the composite material was incubated with SBF. In addition, it was observed that the nHA-reinforced polymer material facilitated an enhanced growth of apatite on the surface, resulting in a decrease in the pore sizes in between the nanofibers, which in turn improved the mechanical strength of the composite with more elongation than the bare PPEES nanofiber. From the results, it was found that the incorporation of nHA considerably influenced the stiffness of the composite material.

### Biomineralization

**Figure 3:**
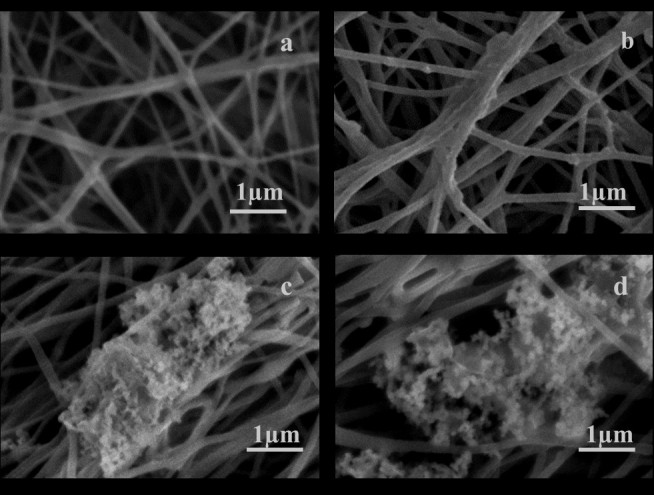
**SEM image showing the surface morphology of PPEES and its composites after incubation in SBF.** (**a**) PPEES after 30 days, (**b**) PPEES 2 after 5 days, (**c**) PPEES 2 after 15 days, and (**d**) PPEES 2 after 30 days of incubation in SBF.

The surface morphology of mineralized PPEES and PPEES 2 composites are shown in Figure 3. The PPEES nanofiber showed no signs of apatite formation even after 30 days of incubation in SBF (Figure 3a). Comparatively, there was significant apatite layer formation on the composite material containing nHA with respect to the incubation time, implying the vital role of nHA in the bioactivity. In addition, it was perceived that in bioactive ceramics, nHA acted as a nucleation site and enhanced the growth of apatite by utilizing the existing ions in the SBF solution. Moderate increase in density of the minerals on PPEES 2 composite after 5, 15, and 30 days of incubation in SBF solution was evident (Figure 3b–e) due to the presence of nHA, and it clearly elucidated that the increased incubation time improved the enhanced apatite formation.

**Figure 4:**
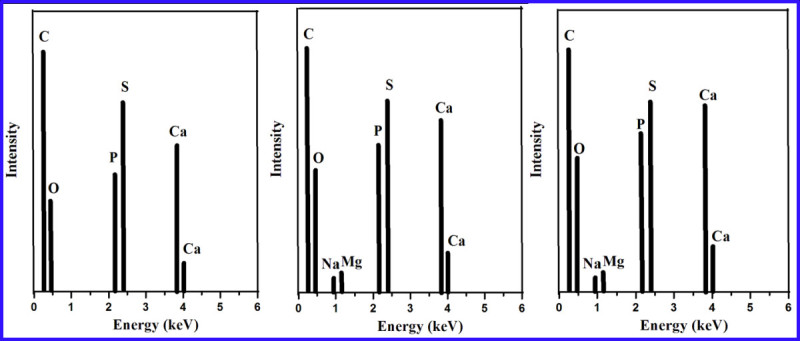
**EDX profile of biomineralization of PPEES 2 composite (a) before, (b) after 15 days, and (c) after 30 days of incubation in SBF.**

Moreover, the mineralization of HA on the polymer nanofiber composite after incubation in SBF was analyzed using EDX, and the spectra were shown in Figure 4. PPEES 2 showed apatite formation over the surface of the nanofiber after 15 days of incubation in SBF (Figure 4b), and interestingly, significant mineralization with dense apatite layer was observed on day 30 (Figure 4c). However, in the case of the bare polymer, the absence of mineralization was obvious. From the results, it was inferred that the polymer nanocomposite reinforced with HA acted as a stimulus for the formation of the apatite layer and was found to be a major factor for mineralization.

Furthermore, the analysis showed that cauliflower-like morphology (Shanmuga Sundar and Sangeetha 2012) of the apatite layer composed mainly of HA, as visualized from SEM identified with peaks of Ca and P elements, with the intensity of the peaks increasing with increase in the incubation time in SBF-endorsed biomineralization (Kim et al. 2004). The Ca and P peaks observed from the EDX analysis were typical of HA. Specifically, *in vitro*, an acellular SBF with ion concentrations nearly equal to those in the blood plasma could reproduce the formation of apatite layer on the polymer nanofiber composite (Kim et al. 2004). From these *in vitro* studies, it was confirmed that the polymer composite reinforced with HA would potentially offer enhanced biomineralization after implantation *in vivo*.

### Cell viability

**Figure 5:**
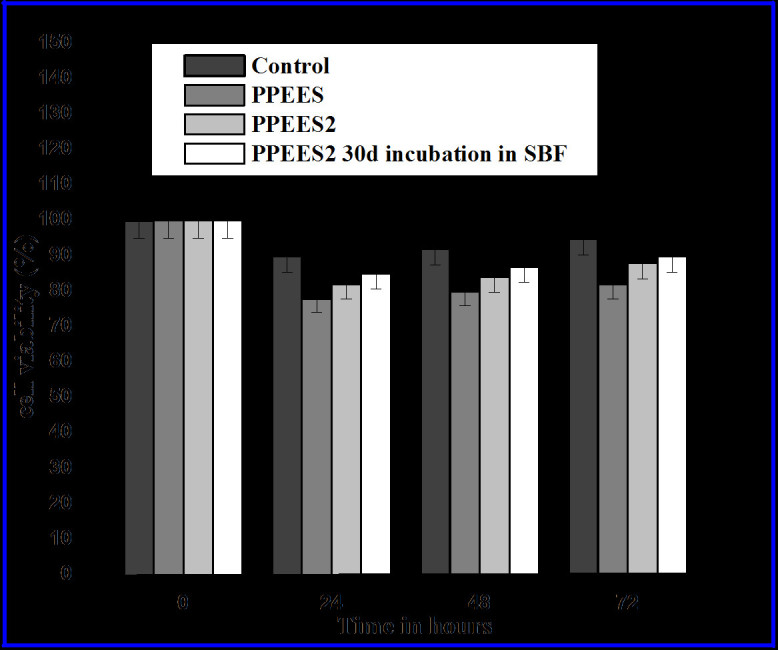
**Percentage viability of osteoblast (MG-63) cells on PPEES 2 nanofiber composites.**

Figure 5 shows the viability of osteoblast-like cells after being cultured with PPEES 2 composites at different time intervals. Good cell adherence on the electrospun nanofibers might be due to the large surface area available for cell attachment (Bhattarai et al. 2005). It is well known that the rough surface formed by the incorporation of nHA on the polymer composite considerably favors cell adherence. In the present study, the optical density value obtained for the control was taken as 100%. The percentage viability of cells on the PPEES 2 composite was extensively similar to the control with different periods of time, as shown in the results of the test in Figure 5. In addition, it was noted that the percentage viability of cells observed with PPEES did not differ significantly from that observed for PPEES 2. With the bioactivity of the composite, rendered by the bioactive component, nHA would facilitate tissue (bone) growth adjacent to the implant and thus helping in the osseointegration of the implant *in vivo* (Wang 2003).

### Cell morphology

**Figure 6:**
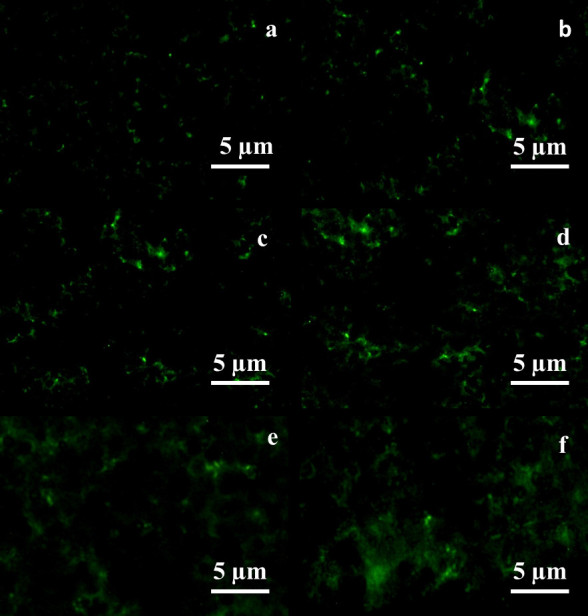
**Inverted fluorescence microscopy images after 3-, 7-, and 10-day cultures of PPEES nanofiber (a–c) and PPEES2 nanofiber (d–f) composites.**

The inverted fluorescence microscopy observation shows the adherence morphology of osteoblast-like cells over the PPEES/nHA nanofiber composites after different culture periods. During the culture period, seeded cells get adhered and proliferated on the fiber composite with apatite formation than with bare nanofiber composite. This may likely be due to higher cell adhesion on apatite-formed PPEES nanofiber composite. This is in agreement with earlier studies (Kang et al. 2008). PPEES 2 nanofiber composite showed more adherences with enthusiastic migration of cells as inferred with fluorescein dye penetration through the osteoblast cell membrane (Figure 6d–f). The intensity of fluorescence on the nanofiber composite increased with the increase in culture time representing the enhanced proliferation of MG-63 cells. From the figure, it is further inferred that the void space in between the nanofibers in the composite was packed with bone-like cells as evidenced by greater cytocompatibility of the apatite-reinforced nanofiber composite. These findings were in line with those observed by Peter et al. (2010) in their studies on chitosan-gelatin/nanohydroxyapatite composites. In the unreinforced chitosan-gelatin (CG) scaffolds, only few cells (osteoblasts) were observed, and the cell morphology was described as rounded. In the case of CG/nHA composite scaffolds, greater cell attachment and spreading were noted while the cell morphology was described as flattened and sheetlike with filopodial extensions. This change in morphology was observed because nHA apparently improved the formation of focal adhesion and allowed for substantial cell spreading. This is quite likely related to the enhanced protein adsorption on the surface in the presence of nHA.

### Cell differentiation: ALPase activity

**Figure 7:**
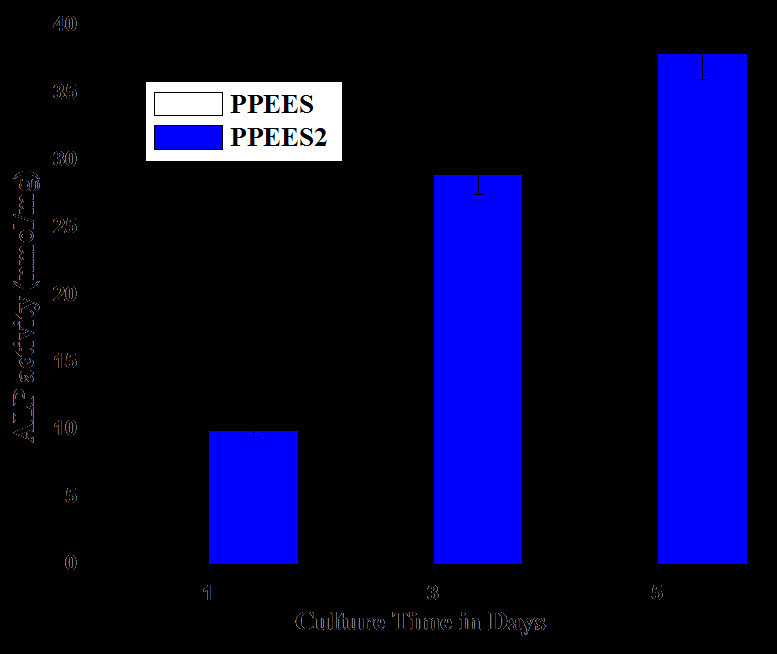
**Cell differentiation of osteoblast on PPEES nanofiber and its composite.**

The ability of cells to differentiate on the surface of the composite after implantation was identified using alkaline phosphatase (ALP)ase activity. Figure 7 showed the osteogenic activity of the polymer composite at different culture time intervals. The differentiation of cells on both bare and nHA-incorporated PPEES nanofibers was increased significantly after 1 day of culture. Surprisingly, the rate of differentiation on bare PPEES nanofiber composite was reduced with respect to different periods of culturing time when compared with PPEES nanofiber reinforced with nHA. Rough surface offered by the apatite layer formed over the nanofiber provided the opportunities for cell adhesion and resulted in an increased rate of proliferation and differentiation. After 3 and 5 days of culture, the color intensity of the cell suspension was found to increase with the addition of alkaline phosphatase which led to the inference of higher cell differentiation in the PPEES 2 nanofiber composite. In the case of bare PPEES nanofibers, the color change was mild, reflecting the low differentiation of MG-63 cells. From the results, it was found that progress in the biomineralization of nanofiber composites significantly encouraged the differentiation of osteoblast cells, which led to bone formation (Dong et al. 2010). Similar studies performed by Srinivasan et al. (2012) using biocompatible alginate/nanobioactive glass ceramic composite scaffolds showed that ALP activity increased up to 7 days and then decreased, indicating the completion of osteoblastic differentiation.

## Conclusions

The PPEES nanofiber composite was successfully fabricated using electrospinning technique. The FTIR-ATR study revealed the presence of HA in the PPEES polymer composite. The SEM images of the nanofiber composites confirmed the formation of beads when nHA was above 5 wt. %. The incorporation of nHA in PPEES showed substantially improved biomineralization and osseointegration with greater bone-forming ability *in vitro.* In addition, the apatite formation on the surface of the nanofiber mat after incubation in SBF solution revealed better bioactivity and mechanical property of the composite when compared with the PPEES nanofiber mat. Moreover, elongation of the PPEES 2 composite significantly amplified with respect to apatite formation *in vitro* evidences adequate mechanical property of the composite when implanted *in vivo.* Furthermore, the viability of cells was observed to be higher in the composite with apatite formation, suggesting the affinity of the composite to the natural hard tissues. Hence, it was concluded that the distinctive features of the composite material would play as an ideal candidate for orthopedic application, furthermore in the replacement of hard tissues.

## Methods

### Materials

Organic polymer, PPEES (CAS number 28212-68-2), and inorganic filler, HA nanopowder (CAS number 12167-74-7), were procured from Sigma-Aldrich Corporation, St. Louis, MO, USA. The solvent used for this study was reagent grade *N*-methyl pyrrolidone (NMP), which was purchased from Merck-India Ltd, Mumbai, India. The bone-like cells (MG-63) used to study the viability of composite materials were acquired from National Center for Cell Sciences, Pune, India. The ingredients utilized for culturing cells such as Dulbecco’s modified Eagle’s medium, fetal bovine serum (FBS), and penicillin-streptomycin were purchased from HiMedia, Mumbai, India. The diagnostic kits such as MTT and ALP were obtained from Sigma-Aldrich Corporation.

### Preparation of nanofiber composite

Various concentrations of PPEES and nHA were used to fabricate the composites to identify their potential for orthopedic applications. The viscous solution was prepared by dissolving PPEES in NMP, and the filler, nHA, was incorporated into it in different weight percentages mentioned in Table 2. The whole content was kept under magnetic stirring overnight and then subjected to ultrasonication for 30 min in order to disperse the nHA uniformly in the solution prior to the start of the electrospinning process. The size of the nHA incorporated into the polymer matrix was around 80 nm as reported through TEM analysis in our previous study (Kalambettu et al. 2012).

**Table 2:** **Different composition of nHA-reinforced PPEES nanofiber composite**

Named composites	Prepared composites	PPEES (g)	nHA (g)
PPEES	PPEES	10	0
PPEES 1	PPEES/nHA (2.5 wt.%)	9.75	0.25
PPEES 2	PPEES/nHA (5.0 wt.%)	9.5	0.5
PPEES 3	PPEES/nHA (7.5 wt.%)	9.25	0.75

The polymer solution containing different concentrations of nHA was loaded into a 2-ml syringe which was linked to a power supply that was capable of generating high voltage up to 50 kV. The flow rate of the syringe pump was regulated using the PICO Espin 2.0 version software. Electrospinning was performed with an electric voltage supplied at 25 kV with a needle tip to a collector distance of 20 cm. The flow rate was adjusted to 0.2 ml/h, and the collecting drum was regulated to rotate at a speed of 1,000 rpm.

A good fiber mat is one which is either devoid or has minimal bead formation (Huang et al. 2003). From the different weight percentages of prepared nanofiber composites, PPEES/5 wt.% nHA (PPEES 2) composite, showed better fiber formation and were used for further investigations of compatibility and mechanical properties for orthopedic applications.

### Characterization studies

#### FTIR-ATR

The composite samples were subjected to FTIR analysis using Alpha T Bruker Optics FTIR spectrophotometer (BRUKER, Billerica, MA, USA). The functional groups present in the polymer and the interaction between the polymer/nHA nanofiber composites were measured using ATR. The frequency of each sample was recorded at a resolution within the scanning range of 4000–500 cm^−1^.

#### SEM

The morphology and dispersion of particles in the polymer matrix were observed using HITACHI S-3400 model SEM (Hitachi High-Tech, Minato-ku, Tokyo, Japan). The surface of the materials was sputter-coated with gold before being subjected to SEM in order to make them electroconductive. SEM analysis of the cross section of the composites was also done to better visualize the presence of nHA in the fiber matrix.

### Mechanical properties

The ability to resist breaking under tensile stress is one of the most important and widely measured properties of materials used in structural applications. The mechanical properties of the nanofiber and its composite were observed using a universal testing machine (UTM). Tensile testing was done according to the ASTM D638 type 5 standard using Hounsfield UTM with a crosshead speed of 2 mm/min and maximum load of 500 N. The percentage elongation of the nanofiber composites was calculated using the following formula:

1 $$\text{Percentage}\;\text{elongation}=\frac{L_{x}-L_{0}}{L_{0}}\times 100\text{,}$$

where *L*_*x*_ = final gage length and *L*_0_ = initial gage length.

Tensile strength can be calculated based on the following formula:

2 $$\text{Tensil}\;\text{Strength}=\frac{F}{A}\;\left(N/mm^{2}\right)\text{,}$$

where F = force (N) and *A* = cross-sectional area (mm^2^).

### Biomineralization

Biomineralization of the polymer nanofiber and its composite with HA was evaluated by analyzing the HA layer formed on the surface of the samples after 15 and 30 days of incubation in SBF solution, which was prepared in the laboratory according to the procedure developed by Kokubo (Leonor et al. 2007). The samples were then retrieved, dried in an oven at 40°C for 4 h, and then examined under SEM-EDX.

### Cell viability

Cytotoxicity studies using the composite nanofiber were carried out on 96-well plates using osteoblast cell lines (MG-63) by MTT assay (Mossman 1983; Sgouras and Duncan 1990). MG-63 cell lines were cultured using Dulbecco’s modified Eagle’s medium (HiMedia), supplemented with 5% FBS and 1% penicillin-streptomycin, and then seeded into the 96-well plate. The wells were sterilized with 70% ethanol followed by UV treatment for 4 h and were neutralized with a phosphate buffered saline (PBS) (pH 7). The wells without the polymer samples were the control groups for the experiment. The MG-63 cell lines were seeded at a density of 6–7 × 10^3^ cells per well and incubated at 37°C in a humidified atmosphere containing 5% CO_2_. In all culture conditions, the medium was renewed every 48 h. After 3 days of incubation, the supernatant of each well was removed and washed with PBS. MTT, diluted in serum-free medium, was added to each well, and the plates were incubated at 37°C for 3 h. After aspirating, the MTT solution, acidified isopropanol (0.04 N HCl in isopropanol), was added to each well and pipetted up and down to dissolve the dark blue formazan crystals and then left at room temperature for a few minutes to ensure the dissolution of all crystals. Finally, the absorbance was measured at 570 nm using an ELISA reader. The viability of cells on the composites was visualized using an inverted fluorescence microscope (Nikon Eclipse, TE 300, Nikon Co., Shinjuku, Tokyo, Japan) after different culture periods (Liu and Tirrell 2008). The composites were transferred to a fresh medium containing 50 ng/ml fluorescein diacetate, incubated for 5 min, and then washed with PBS. The membranes of viable cells, penetrated with the dye solution, were then excited at 488 nm under the inverted fluorescence microscope.

### Cell differentiation: ALPase activity

Differentiation of osteoblast cells on the nanofiber composite reinforced with nHA was assessed *in vitro* by ALP activity. Many scientists rely upon the ALP activity of osteoblasts as an indicator of their degree of differentiation (Sila-Asna et al. 2007; Thangakumaran et al. 2009). In the present study, a calorimetric assay was used to find the ALPase activity, where the release of yellow-colored *p*-nitrophenol from *p*-nitrophenol phosphate substrate was monitored by measuring the optical density at 405 nm. Sigma diagnostic kit number 104 was used to estimate the ALPase activity of the cells.

### Statistical analysis

Quantitative data were expressed as mean ± standard deviation. Statistical analysis was carried out using ANOVA test. Statistical significance was set at 0.05, and Origin version 6.0 software was used.

## Authors’ original submitted files for images

Below are the links to the authors’ original submitted files for images. Authors’ original file for figure 1 Authors’ original file for figure 2 Authors’ original file for figure 3 Authors’ original file for figure 4 Authors’ original file for figure 5 Authors’ original file for figure 6 Authors’ original file for figure 7 Authors’ original file for figure 8
