# Supplementary figures and images for: Evaluation of polyphenylene ether ether sulfone/nanohydroxyapatite nanofiber composite as a biomaterial for hard tissue replacement
Source: Prog Biomater. 2013 Feb 6;2:2. doi: 10.1186/2194-0517-2-2 (PMC5120667; doi:10.1186/2194-0517-2-2)

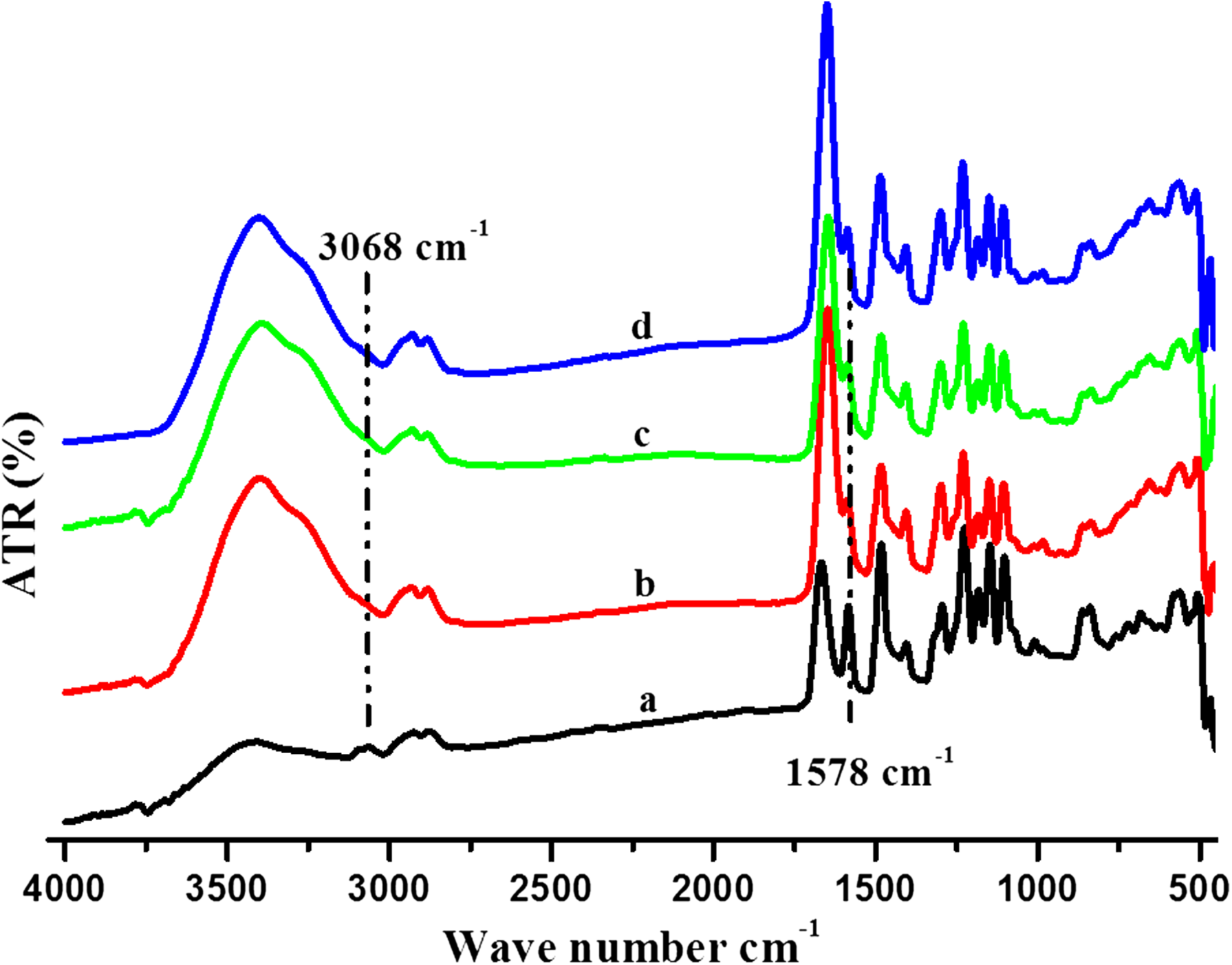

Supplement: Supplementary file 1 — Authors’ original file for figure 1 [file 40204_2012_5_MOESM1_ESM.tiff]

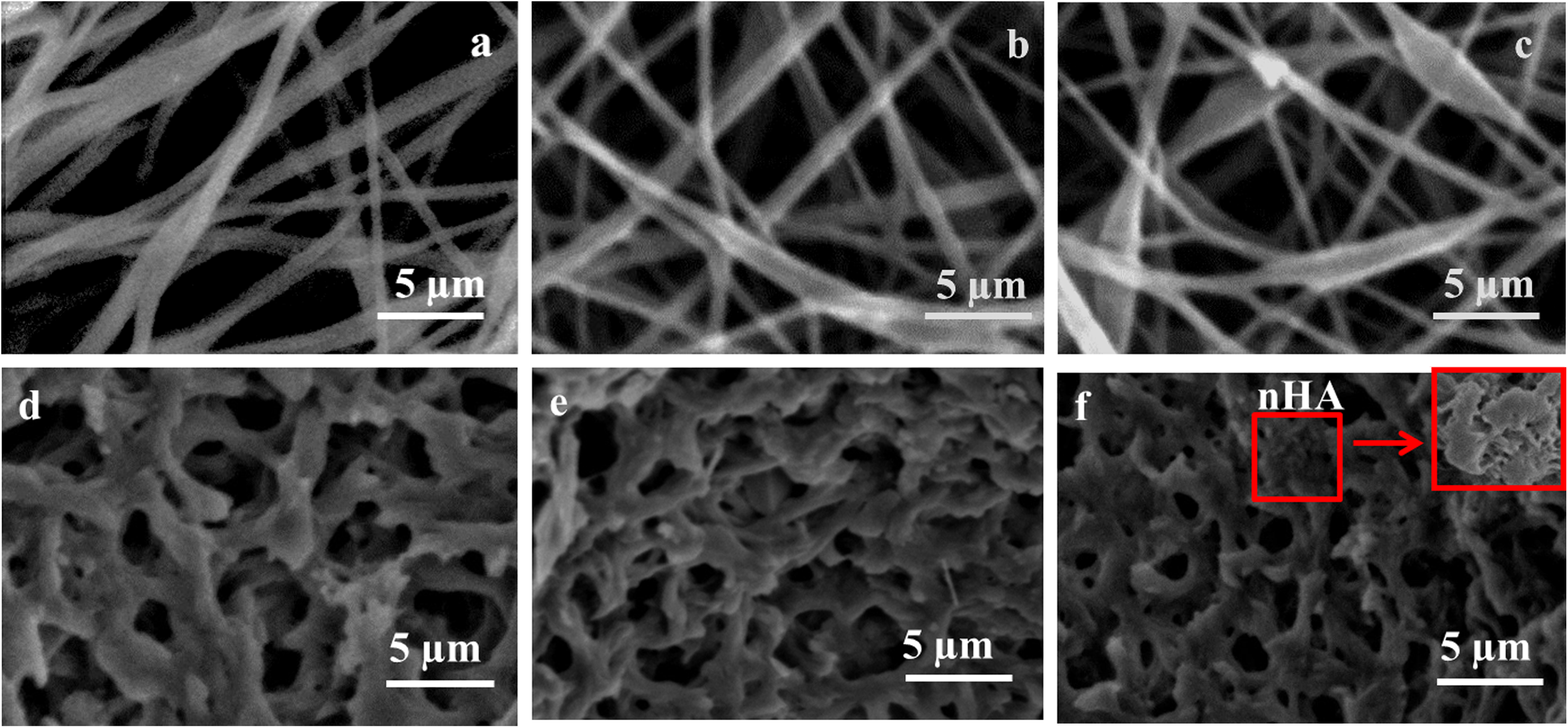

Supplement: Supplementary file 2 — Authors’ original file for figure 2 [file 40204_2012_5_MOESM2_ESM.tiff]

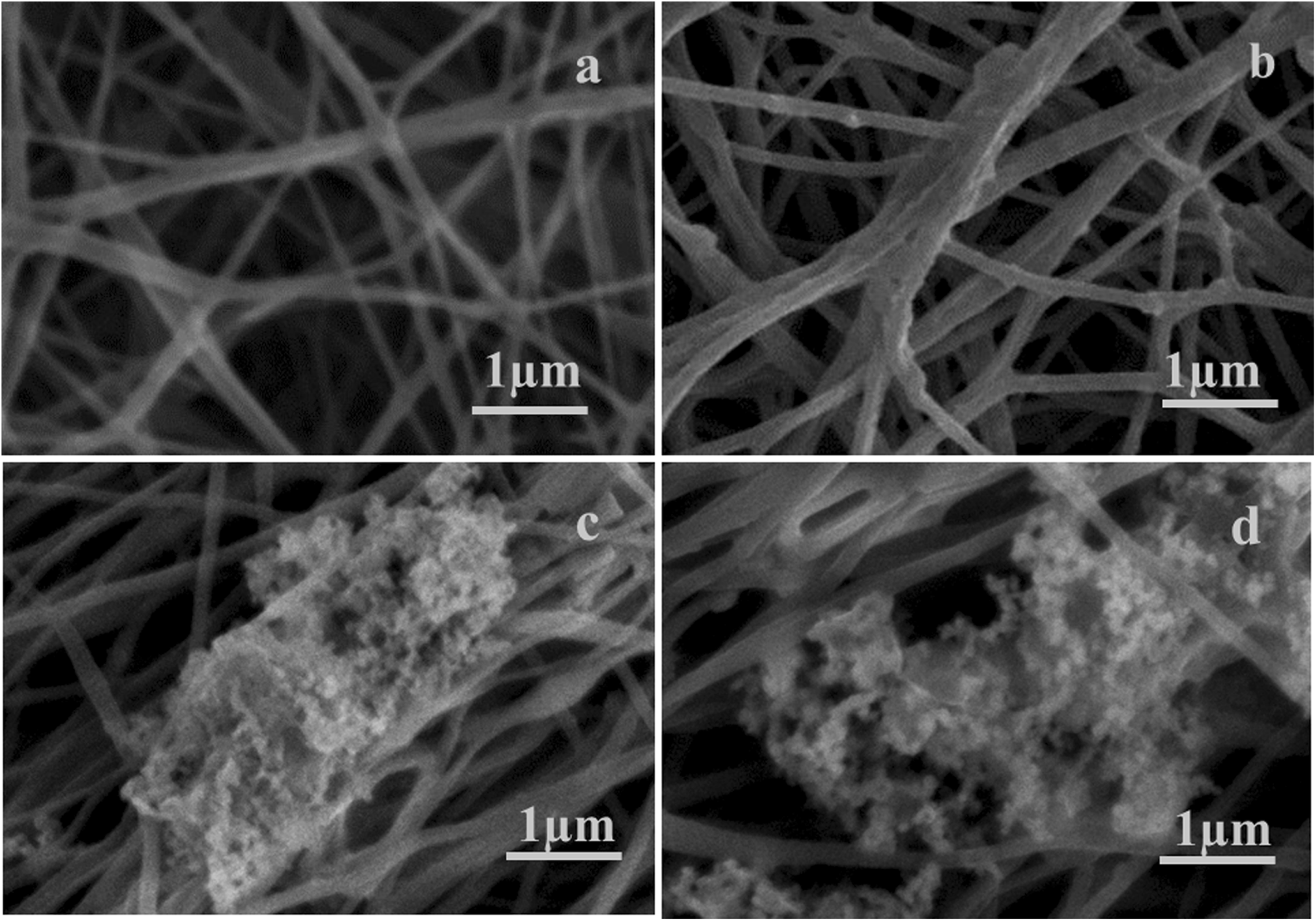

Supplement: Supplementary file 3 — Authors’ original file for figure 3 [file 40204_2012_5_MOESM3_ESM.tiff]

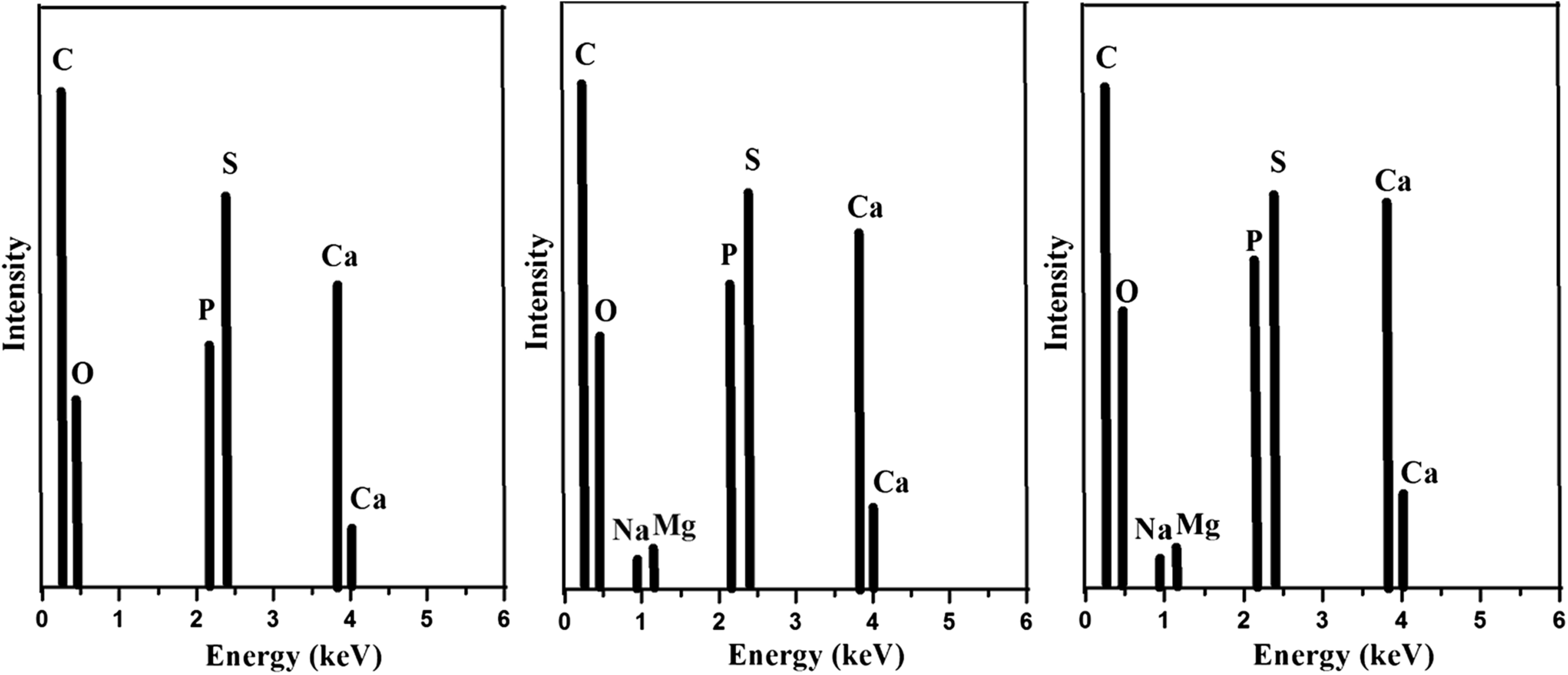

Supplement: Supplementary file 4 — Authors’ original file for figure 4 [file 40204_2012_5_MOESM4_ESM.tiff]

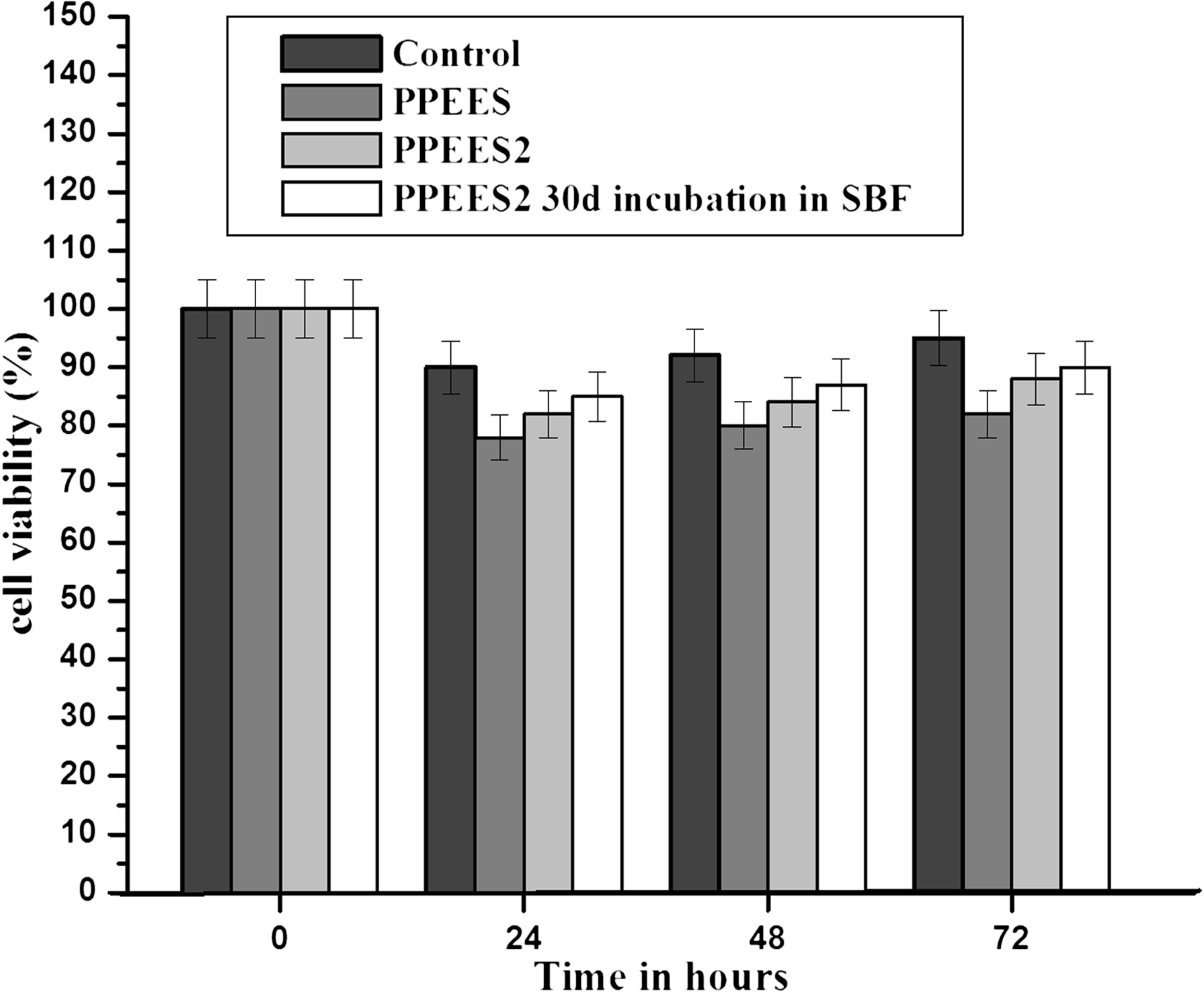

Supplement: Supplementary file 5 — Authors’ original file for figure 5 [file 40204_2012_5_MOESM5_ESM.tiff]

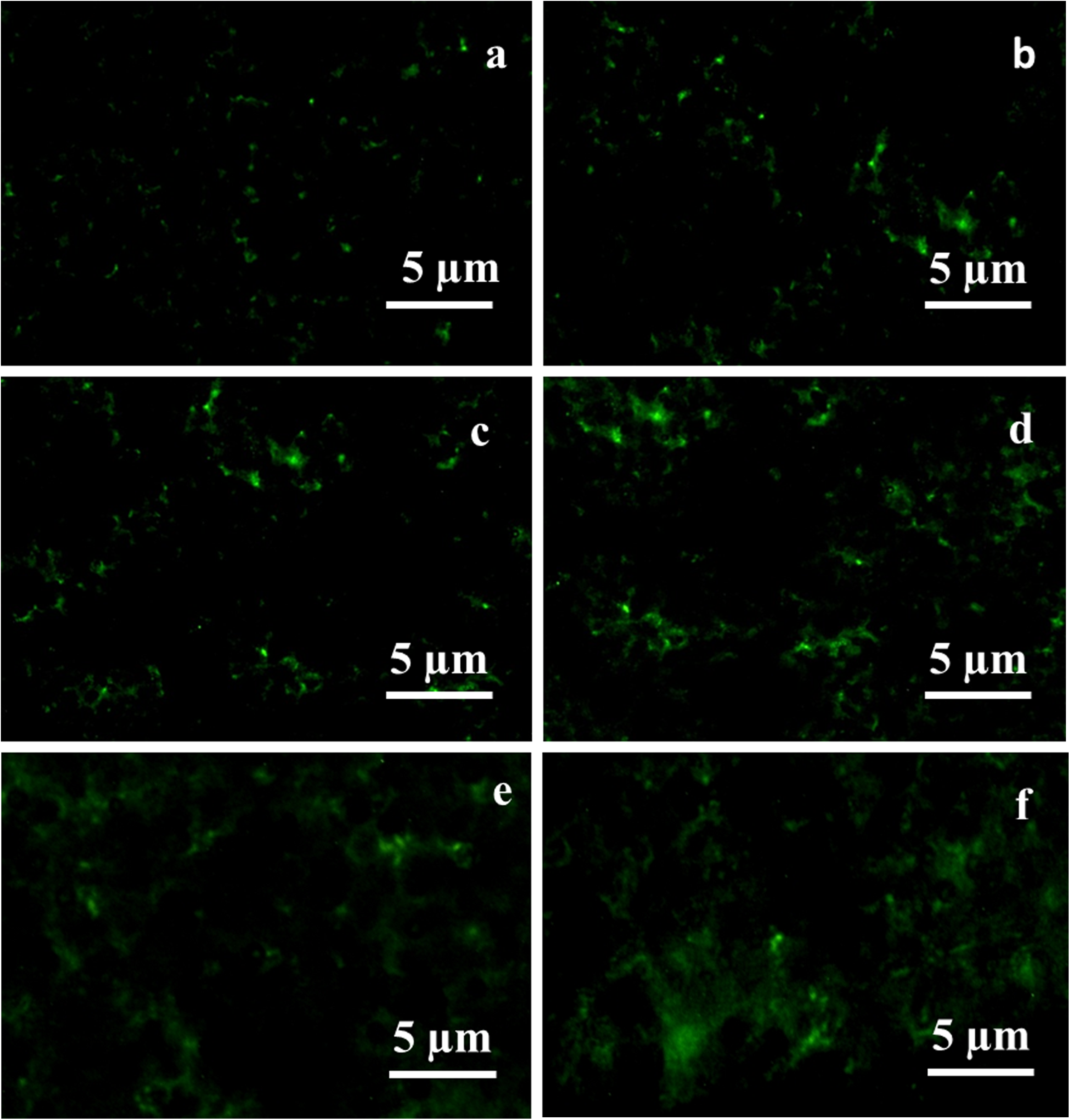

Supplement: Supplementary file 6 — Authors’ original file for figure 6 [file 40204_2012_5_MOESM6_ESM.tiff]

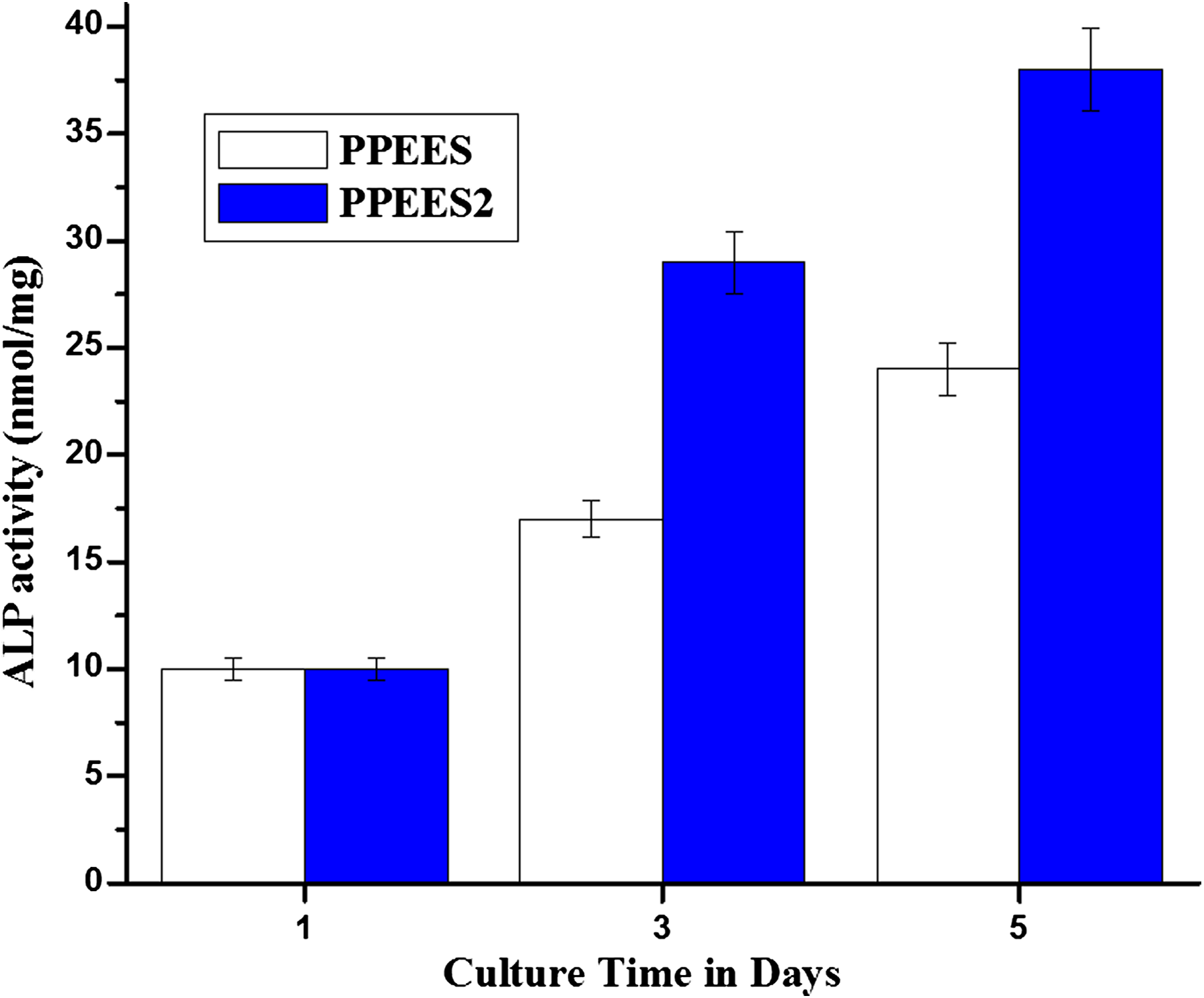

Supplement: Supplementary file 7 — Authors’ original file for figure 7 [file 40204_2012_5_MOESM7_ESM.tiff]

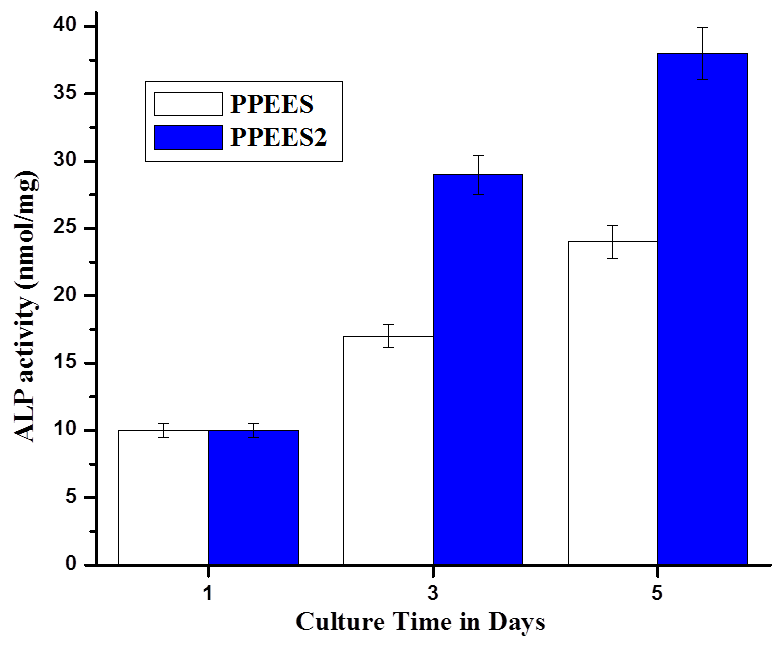

Supplement: Supplementary file 8 — Authors’ original file for figure 8 [file 40204_2012_5_MOESM8_ESM.tiff]
